# Editorial: Ergogenic Aids: Physiological and Performance Responses

**DOI:** 10.3389/fspor.2022.902024

**Published:** 2022-05-16

**Authors:** Gustavo R. Mota, Moacir Marocolo

**Affiliations:** ^1^Exercise Science, Health and Human Performance Research Group, Department of Sport Sciences, Institute of Health Sciences, Federal University of Triângulo Mineiro, Uberaba, Brazil; ^2^Physiology and Human Performance Research Group, Department of Physiology, Federal University of Juiz de Fora, Juiz de Fora, Brazil

**Keywords:** enhancement, exercises, physical training, sports and exercise medicine, recovery

## Introduction

Regarding exercise and sports science for performance, ergogenic aids are substances, devices, techniques, or phenomena that are work-producing and are believed to increase performance (and or recovery), directly or indirectly (da Mota and Marocolo, 2016; Arriel et al., 2018). Multiple categories of ergogenic aids aim to enhance exercise or sports performance and have typically been categorized by their pharmacological, physiological, nutritional, mechanical, or psychological mechanisms (Bishop, 2010; Londe et al., 2018; Marocolo et al., 2018, 2019). However, overall, ergogenic aids may not act by one mechanism only. For example, sodium bicarbonate may be classified as a physiological and nutritional aid, as well as a psychological aid. The same rational is applied for compression garments (Gimenes et al., 2019; Pavin et al., 2019) and ischemic preconditioning (mechanical, physiological, and psychological) (Marocolo et al., 2016, 2019; Da Mota et al., 2019).

These strategies may improve energy readiness and/or accelerate the recovery course (chronically or acutely), eventually augmenting performance. Though, many athletes commonly use these ergogenic aids even without any scientific evidence (Bishop, 2010; Marocolo et al., 2018). Therefore, researchers should perform studies meeting the main research design issues, such as dose of the intervention/substance; background of volunteers (e.g., professional; amateur); specific exercise type (e.g., strength, endurance); and well-controlled studies (e.g., placebo; double-blind).

Therefore, the current Research Topic (RT) aimed to present scientific answers from well-controlled research studies that show the effectiveness (or rebut) of ergogenic aids promising to boost exercise or sports performance, emphasizing the physiological mechanisms.

## Articles

This RT includes four original research articles (Clark et al.; Giráldez-Costas et al.; Harty et al.; Martin-Arrowsmith et al.), one brief research report article (Hannaian et al.), and one review article (Arazi and Eghbali). In the single review article, the authors presented potential mechanisms underlying beetroot supplements such as signaling pathways inhibiting inflammatory diseases, improved blood flow, and mitochondrial function (Arazi and Eghbali).

Clark et al. investigated whether exogenous ketone salts alone or combined with whole-body cooling might improve the 30 min steady-state preload cycling, followed by a 15 min time trial, or impact the substrate metabolism. It was concluded that ketone salt supplementation does not influence performance and does not offer a significant change in energy metabolism during exercise (Clark et al.). Another study (Martin-Arrowsmith et al.) that evaluated the effects of ketone monoester supplement on muscle damage during eccentric exercise recovery also failed to report positive findings. Twenty healthy volunteers were tested in a randomized, double-blind independent group design. In short, 360 mg/kg/body mass of ketone monoester did not surpass the effects of energy-matched carbohydrates on jumping, muscle isometric action, or muscle soreness, although peak blood β-OHB concentration was higher (Martin-Arrowsmith et al.).

Caffeine is one of the most studied ergogenic aids. The effects of caffeine intake have also been highlighted in this Research Topic. A randomized double-blind experiment (Giráldez-Costas et al.), involving 16 participants over 4 weeks, tested whether caffeine intake (3 mg/kg/body mass), before each training session, would impact adaptations induced by a bench press training protocol. Their results showed no improvements in the 1 RM test obtained after a 4-week program, yet performance gains were larger as evidenced by higher mean and peak values for both velocity and power over the placebo (Giráldez-Costas et al.). Beyond caffeine use, the time between intake and exercise or testing were also reported. Harty et al. investigated the optimal pre-exercise time interval to consume caffeine to improve lower-body muscular performance, also comparing sex differences. Eighteen healthy volunteers ingested 6 mg/kg 2 h, 1 h, or 30 min before muscle exercise tests. The main result was that caffeine ingestion 1 h before exercise exerts the most consistent ergogenic benefits relative to other time points (Harty et al.).

Because it does not interfere with the performance of training sessions or require almost no extra time outside the routine, protein supplementation is a popular intervention. Hannaian et al. determined whether immediate, compared to delayed, protein ingestion would benefit a greater acute recovery of exercise performance (anaerobic peak power and muscle strength) during subsequent days of variable intensity training (Hannaian et al.). They concluded that successive days of simulated team sport exercise decrease markers of next-day performance with no effects of protein timing on acute recovery. However, the training performed increased muscle strength and aerobic capacity in as little as 5 days with the latter potentially enhanced by immediate post-exercise protein consumption (Hannaian et al.).

## Final Considerations

Although some studies testing nutritional or supplementation strategies have shown beneficial effects on exercise performance and/or recovery, these effects are not universal. The administration of these types of ergogenic aids usually requires more attention, especially regarding time and magnitude effect, and should not be used as the main resource of performance gains.

## Author Contributions

GM and MM conceived the idea, wrote the first draft, worked on all drafts, and formatted the manuscript for submission. Both authors read and approved the last version of the manuscript.

## Conflict of Interest

The authors declare that the research was conducted in the absence of any commercial or financial relationships that could be construed as a potential conflict of interest.

## Publisher's Note

All claims expressed in this article are solely those of the authors and do not necessarily represent those of their affiliated organizations, or those of the publisher, the editors and the reviewers. Any product that may be evaluated in this article, or claim that may be made by its manufacturer, is not guaranteed or endorsed by the publisher.
